# Exploratory structural equation modeling: a streamlined step by step approach using the R Project software

**DOI:** 10.1186/s12888-023-05028-9

**Published:** 2023-07-28

**Authors:** Maria Prokofieva, Daniel Zarate, Alex Parker, Olympia Palikara, Vasileios Stavropoulos

**Affiliations:** 1 Institute for Health and Sport, Victoria University, Melbourne, Australia; 2 School of Health and Biomedical Sciences, RMIT University, Carlton, Australia; 3 Department for Education Studies, University of Warwick, Coventry, UK; 4 Department of Psychology, National and Kapodistrian University of Athens, Athens, Greece

**Keywords:** Exploratory Structural Equation Modeling, ESEM, R software, Mplus, Syntax, Longitudinal Study of Australian Children

## Abstract

Confirmatory Factor Analysis (CFA) has been a popular yet limited approach to assessing latent factor structures. Despite items rarely loading exclusively on one latent factor in multifactorial scales, CFA assumes all indicators/items should load uniquely on their allocated latent dimensions. To address this weakness, Exploratory Structural Equation Modeling (ESEM) combines exploratory factor analyses (EFA) and CFA procedures, allowing cross-loadings to occur when assessing hypothesized models. Although such advantages have enhanced ESEM popularity, its adoption is often limited by software rigidity and complex coding difficulties. To address these obstacles, the current tutorial presents a streamlined, step-by-step approach using the open-source software R while providing both R and Mplus ESEM syntax. The tutorial demonstrates the sequence of the ESEM stages by examining the frequently debated Strengths and Difficulties Questionnaire (SDQ) factor structure, using openly accessible data from the Longitudinal Study of Australian Children (LSAC). As ESEM may allow a better understanding of the complex associations in multidimensional scales, this tutorial may optimize the epidemiological and clinical assessment of common yet multifaceted psychiatric presentations.

**Supplementary Information:**

The online version contains supplementary material available at 10.1186/s12888-023-05028-9.

Human behavior researchers commonly employ self-report scales to assess individuals’ experiences, including complex psychiatric presentations [1, 2]. Such instruments usually include items or indicators assumed to capture latent constructs [3]. Latent factors are then identified to account for the correlations among indicators (i.e., items influenced by the same underlying construct), and potential combinations of latent factors result in latent factorial structures [4]. However, given that items and instruments often represent poor modeling of chosen phenomena, psychometric analysis and validation are imperative [5, 6]. Considering potential limitations of traditional exploratory and confirmatory approaches, the present paper aspires to introduce a novel, automated and freely accessible exploratory structural equation modeling (ESEM) tutorial using the R software. Moreover, this paper provides a practical example using a widely employed assessment scale. The results of the method are comparatively examined with those of other popular ESEM calculation approaches.

## What are the limitations of commonly applied validation approaches?

Depending on whether a researcher primarily explores or confirms a hypothetical factor structure, different analysis options are available to guide the revision and improvement of a questionnaire [4]. Accordingly, Exploratory Factor Analysis (EFA) is data-driven, and no prior specifications may be made regarding the number of factors or pattern(s) of indicator/factor relationships. Alternatively, Confirmatory Factor Analysis (CFA) assumes a-priori item-factor loadings/associations and constraints estimation of others [6]. Moreover, both EFA and CFA help understand item functioning, and thus have been extensively implemented in applied research. However, these methods may have substantial limitations [7]. Although EFA enables researchers to obtain the optimal factorial structure of a scale based on the extraction of common items’ variance, the dimensions extracted might not always be theoretically meaningful and thus useful for comparisons across groups and over time [8, 9]. Furthermore, in EFA, correlations between pairs of items due to specific methodological influences (e.g., similar language delivery) are only considered in the context of residual variance(s) [9]. Thus, the exploratory stage (i.e., EFA) usually requires a confirmation or validation stage (i.e., CFA) [8, 9].

Questionnaire validation via CFA can be challenging in the case of multifactorial structures, where hypothesized (not explored) structures propose congeneric items (i.e., loading on only one factor) [6]. However, items often relate to factors other than the primary item-allocated ones (i.e., cross-loadings [8]). Cross-loadings are rarely equal across items and should be modeled to increase measurement validity [6]. CFA approaches ignore cross-loadings, prioritizing parsimonious models that may result in limited model fit and measurement indices (e.g., reliability [9, 10]). Moreover, not accounting for minor cross-loadings can generate reduced fit for theoretically sound instruments with a larger number of factors (e.g., 5–10) and a high number of items (e.g., 5–10/ per scale/dimension [11]). Thus, ignoring potential item cross-loadings inevitably affects the validity and utility of CFA findings [9].

## Exploratory Structural Equation Modeling (ESEM): How is it similar and different to traditional EFA and CFA procedures?

Alternatively, Exploratory Structural Equation Modeling (ESEM) integrates EFA and CFA strengths to overcome their limitations [7–10, 12]. ESEM is an EFA conducted in a structural equation modeling (SEM) context where items can load on multiple factors and produce goodness-fit indices (see Table 1). Thus, ESEM has been suggested to merge the advantages of both EFA and CFA analyses [7]. Consequently, ESEM considers items’ cross-loadings as little as 0.10 and/or even approximating 0, preventing inaccurately increased parameters or distorted model-fit [13]. It should be noted, however, that ESEM may not work best in bifactor structures (i.e., latent structures where each item loads on specific uncorrelated factors as well as a general common factor [12]). With this in mind, ESEM calculations with target rotation have been introduced, enabling cross-loadings to be embedded within hypothesis-derived models (i.e., targeted items are considered for both their targeted and non-targeted dimensions [8, 10, 14–16]). This type of rotation also “targets” cross-loading to approximately zero for non-primary item-factor associations [16].

**Table 1:** Comparison of EFA, CFA and ESEM

	**EFA**	**CFA**	**ESEM**
Theory/Data	Data driven approach, (exploratory)	Theory-driven, (confirmatory)	Fundamentally, confirmatory technique that integrates exploratory elements
Item-Factor Loadings	Cross-loadings are allowed and not fixed. loadings are freely estimated	Cross-loadings are not allowed Unique specification of items onto respective latent factors applies	Non-target cross-loadings are constrained to be as close to zero as possible, but are still allowed
Factor Structure & Parsimony	Complex factorial structures may emerge. Issues with parsimony may be present	Parsimonious models. Simple/clear factorial structures (sometimes criticised as overly simplistic)	Complex factorial structures, especially, in large datasets. However, more control applies compared to EFA
Interpretability Risks	Extracted factors may not always be meaningful	Despite the increased insight into scale-scoring, adequate item loadings and high levels of reliability it provides, positively biased factor correlations and lower goodness of fit may be present	With non-zero cross-loadings, the bias and inflated statistics are reduced

For a more in-depth discussion of EFA, CFA and ESEM differences and sum-scores interpretation see [12] and [17]

## What are the different ways to implement ESEM?

Overall, ESEM involves a mixture of exploratory and confirmatory elements, including a) the factorial structure of a scale; b) primary factor loadings, and c) non-primary factor loadings. Such choices of structural ESEM components may later be expanded via the selection of different rotation types and estimators informing both the similarities and the differences between two traditional ESEM pathways, as well as two recently introduced ESEM variations (i.e., set-ESEM; ESEM within CFA [7, 16, 18, 19]. The two alternative, yet similar, traditional ESEM pathways involve a) expanding CFA via EFA features (1^st^ pathway) and b) expanding EFA via incorporating CFA structures/features (2^nd^ pathway). Pathway 1 either expands CFA-calculated models by constraining all cross-loading thresholds to near zero (~ 0) for non-primary items (pathway 1a; see the default Mplus procedure [20]) or by assuming EFA-derived loadings as the threshold for primary loadings and cross-loadings (pathway 1b [19]). Alternatively, the second pathway includes a two-stage process. It initially uses factor analysis to identify the primary items assumed to be allocated to each dimension (or factor). The second stage includes non-primary items, with their latent extracted factors correlated under an ESEM solution. The major difference between pathway 2 and pathway 1b is using EFA procedures as the core of calculations instead of CFAs [18].

In that line, two significant methodological ESEM variations have also been suggested [7, 16]. Firstly, in cases where theoretical arguments support only a number/set of interrelated factors (and not all factors of a multidimensional scale associating with each other) only cross-loadings within this set may be enabled (see set-ESEM method [7]). Secondly, the ESEM-Within-CFA (EWC) has been proposed to compensate for the limited interpretability likely resulting from traditional ESEM [16]. In essence, EWC uses ESEM-item loadings to inform starting values of item loadings in a CFA model, combined with a number of fixed parameters for convergence [16]. Specifically, a scaling/referent item is chosen per factor to help detect small cross-loadings, which are then fixed to their previously ESEM-derived values [16]. All other parameters adhere to traditional ESEM management (i.e., relaxed and/or constraint [16]). Both ESEM variations (EWC and set-ESEM) have been shown to operate well in most cases, despite potentially resulting in weaker performance in ESEM models involving higher-order factors [16].

These different ESEM approaches can be later enhanced with the choice of specific rotation types and estimators (see Table 2). Such choices may have significant effects on the modeling flow (i.e., convergence/parsimony) and the results [12]. Considering estimators in the traditional CFA and EFA context, Maximum Likelihood (ML) estimation tends to be the most widely used for data assuming multivariate normality (commonly involving continuous variables), while Weighted Least Squares (WLS) is the estimator of choice for non-continuous variables/data (i.e., Likert scales [4]). Robust variations of such estimators may also be selected in ESEM modeling to consider standard errors influences in the reported statistics (see Maximum Likelihood with Robust Standard Errors [MLR] and/or Weighted Least Square Mean(s) and Variance(s) adjusted [WLSMV] estimators [4, 21, 22]. Considering rotations, oblique types tend to be more commonly employed in traditional CFA/EFA procedures, as dimensions of multi-factorial questionnaires/scales are often expected to be correlated [4, 21, 22]. However, a series of other alternative options may also be used to expand ESEM calculations based on the specific modeling features (see Table 2 for more details).

**Table 2:** ESEM Pathways 1 & 2, structural features, estimators and rotations

	**ESEM Pathway 1 / Mplus Software**	**ESEM Pathway 2/ R Software**
**Factor Structure﻿**	The first pathway addresses the scale’s factor structure primarily in a confirmatory manner [19]	The second pathway addresses the scale’s factor structure primarily in an exploratory manner [17]
**Primary Loadings**	The first pathway allows items’ loadings to their primary allocated factors to be freely estimated [18, 19]	The second pathway first conducts factor specific EFAs to estimate items’ loadings to their primary allocated factors [17]
**Non-primary Loadings/ Cross-loadings**	In pathway 1a non-primary loadings/ cross-loadings are uniformly addressed with calculation thresholds to near zero values (~ 0) [18, 19] In pathway 1b non-primary loadings/cross-loadings are addressed with the allocation of thresholds extracted after EFA procedures [18]	Factor-specific EFAs are at a second stage expanded to include non-primary items to estimate cross-loadings/non-primary loadings [17]
	**Estimator Type**	**Application**
**Estimator Types Employed Across ESEM Pathways**	Maximum Likelihood [ML]	ML is a generic estimator assuming multivariate normality of the data and thus, normal distribution of eigenvalues. Its general use is recommended for continuous variables, though it can handle categorical or ordered variables but require numerical integration
	Unweighted Least Squares [ULS] / Weighted Least Squares [WLS]/ Diagonal Weighted Least Squares [DWLS]	ULS, WLS and DWLS are recommended for singular or non-positively semi-defined correlation matrices, assuming that the number of factors is less than the matrix rank. They minimize the residuals between the input correlation matrix and the reproduced factorial correlation matrix. ULS, WLS and DWLS are recommended for use with ordinal variables. ULS can also be used with all continuous variables with non-missing data
	Generalised Least Squares [GLS]	GLS is based on the same computational approach as ULS but is recommended when the dataset includes highly unique variables (e. g. likely to be weakly aligned with factors). The estimator, in this case, is likely to show a better fit for common variables, which are strongly aligned with the factors. This is based on the computational approach implemented in the estimator that minimises the residuals and weights correlation coefficients differentially. This estimator is used with large datasets, requiring less computation time and computer memory. However, its estimator requires complete data, and thus, in a general case, ML is preferred
	Robust equivalents: Maximum Likelihood Method [MLM], Maximum Likelihood Method with Mean Variance adjusted Satterthwaite approach [MLMVS], Maximum Likelihood Method with Variance adjustment [MLMV], Maximum Likelihood with First-order derivatives [MLF], Maximum Likelihood with Robust standard errors [MLR]	The use of the robust equivalents of the above estimators is driven by the adjustment required for standard errors and reported statistics. The use of particular estimators in this family is driven by specific dataset and reporting statistics required MLM (Satorra-Bentler scaled test), is recommended for continuous variables with non-missing data. MLR (Huber-White standard errors) shows similar performance with non-missing data but is preferred when missing data and/or non-normality is present. Both MLR and MLF can be used with continuous, unordered and ordered categorical variables but require numerical integration The use of other robust estimators is specific to the required adjustment to the chi-square distribution through chi-square test statistic and/or its degrees to freedom. See Santora and Bentler [20] for details Further details on chi-square difference testing between MLM, MLR and WLSM is described on the Mplus website https://www.statmodel.com/chidiff.shtml
	Weighted Least Squares Mean [WLSM], Weighted Least Squares Mean with Mean Variance adjusted Satterthwaite approach [WLSMVS], Weighted Least Squares Mean with Variance adjustment [WLSMV], Unweighted Least Squares Mean [ULSM], Unweighted Least Squares Mean with Variance adjusted Satterthwaite approach [ULSMVS], Unweighted Least Squares Mean with Variance adjustment [ULSMV]	This family of estimators is used for datasets that include categorical or ordered variables [4]. Additionally, these estimators report on CFI, TLI and RMSEA metrics that may be the preferred choice for the researcher [4] While all these estimators can handle categorical and/or ordered variables, the use of WLSMV in such cases is preferred
	**Rotation Type**	**Application**
**Rotation Types Employed** **Across both ESEM pathways**	Oblique rotations: promax, oblimin, simplimax, geomin Orthogonal rotations: geomin, varimax, quartimax, equamax varimin	Oblique rotation approaches assume that factors are correlated, whilst orthogonal rotation approaches assume that factors are uncorrelated. The majority of rotation variations (e.g., goemin, promax etc.) are offered in both oblique and orthogonal formats The general suggestion for selecting rotation is to start with the default oblique rotation (also implemented as a default setting in both the Mplus and R software) and examine correlations among factors. If correlation scores remain relatively low (e.g., below 0.32), the use of orthogonal approaches is recommended [21] When there is an indication of high loadings on more than one factor, Geomin rotation is suggested When a CFA model involves EFA elements (e.g., ESEM models), the use of Target rotation is recommended, since it allows specification of target factor loadings to rotate the factor loading matrix (see current Tutorial below). The general use is to set factor loadings to zero. To ensure model identification, the minimum number of target values is set at m(m-1) for oblique Target rotation and m(m-1)/2 orthogonal Target rotation, where m is the number of factors in the model For results reporting, it also needs to be noted that *promax* and *varimax* do not provide standard errors, while other rotations support this

*Note:* Factorial structure specifies correlations between the items and the factors and is defined with the instrument/dataset and research approach in mind. It is usually considered at the planning stage and includes a consideration for alternative factorial models. The development of the factorial structure and alternative models in either approach is based on theory-informed hypotheses [9]. The definition of the factorial structure is provided in model specification in relevant steps in either approach

### What are the strengths and limitations of ESEM?

Overall, ESEM tends to produce less biased inter-factor correlations and model estimations [9, 12]. In that line, as the magnitude and the precision of both items’ primary loadings and cross-loadings concurrently define ESEM extracted factors, their clarity may be enhanced via less inflated correlations, resulting in more realistic reliability estimates, as well as improved modeling fit, compared to non-ESEM procedures (e.g., α, ω [8, 14, 15]. In addition, given that ESEM can concurrently employ both CFA and EFA methods, ESEM-extracted latent dimensions and general findings tend to be more accurate reflections of reality and, thus, the phenomena underpinning their measuring scales [9, 10]. Furthermore, ESEM latent factors can counterbalance inter-cultural/national differences related to the interpretation of items and item-wording effects [9, 10].

Despite these strengths, notable ESEM limitations may involve reduced/lack of parsimony (i.e., the method can be too flexible), and latent constructs may be difficult to interpret [6, 23]. Additionally, ESEM may underperform in complex models, as a high model fit may interfere with the calculation of higher-order factors (e.g. partial invariance, mediation employed cross-loadings, multi-level, latent class and latent growth curve modeling, commonly used in psychiatric research [9, 23]). Additional limitations are related to the critical importance of the rotation procedures selected, as these may influence the size and direction of latent factor correlations and cross-loadings [10]. Thus, ESEM modeling should not be viewed as an entirely positive procedure without taking into consideration its limitations and specific uses [7, 9, 23].

## When should ESEM be employed?

**Fig. 1:**
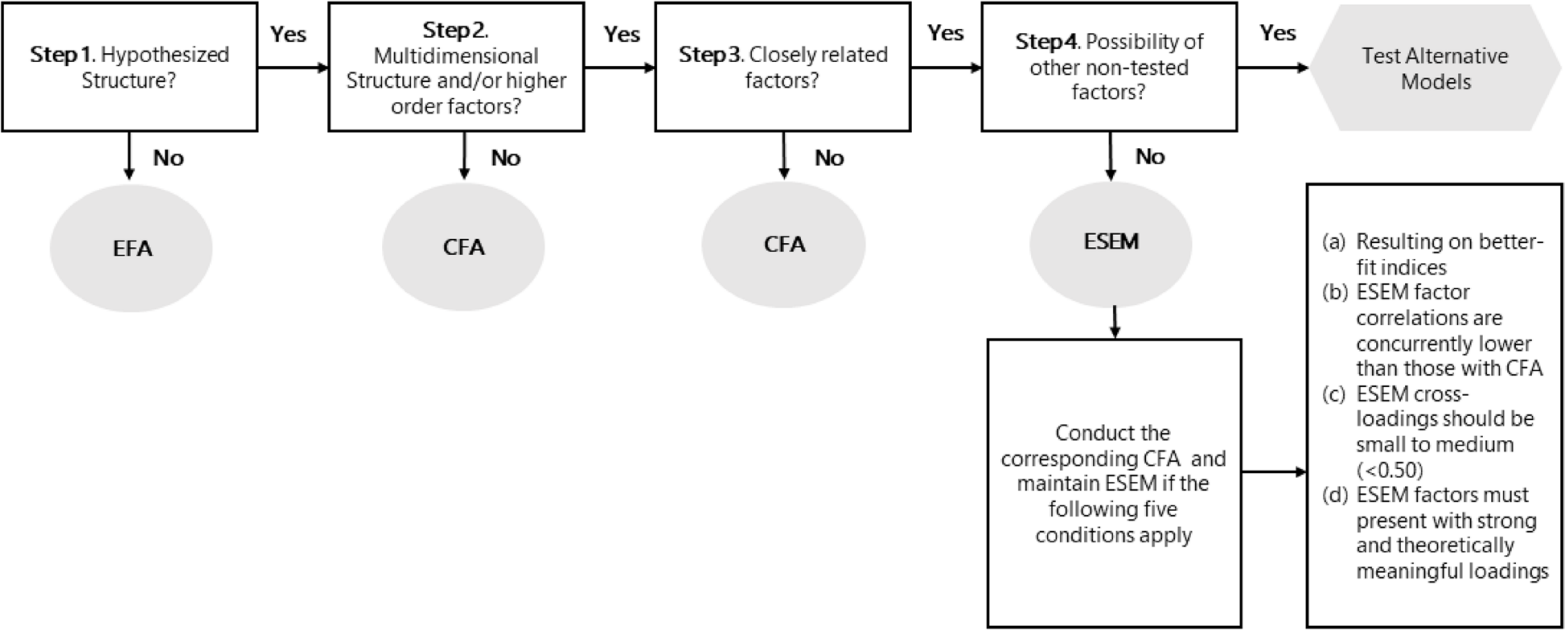
EFA, CFA, ESEM decision process

According to Alamer and Marsh [7], ESEM is a confirmatory procedure enriched via exploratory elements to incorporate non-primary item(s)-factor(s) associations (i.e., cross-loadings). Thus, ESEM is exclusively indicated for multidimensional questionnaires, where an item’s variance may be simultaneously explained by more than one latent factor without necessarily indicating the occurrence of a non-measured alternate factor (e.g., bi-factor models/ non-calculated other factors; see Fig. 1 [7, 10]). Nevertheless, even in the case that all prior conditions apply, ESEM models are recommended to be comparatively calculated with their respective CFA models and preferred only if: a) significantly better fit indices are observed (compared to a CFA model [7]); b) ESEM factor correlations are lower than those of their corresponding CFA estimation; c) ESEM cross-loadings are small to medium (< 0.50) if higher a theoretical (for instance similar item phrasing) explanation applies; d) ESEM factors must present with strong and theoretically meaningful loadings, as medium to large cross-loadings might suggest a non-calculated factor; e) ESEM bi-factor models need to show better fit than its corresponding non-bi factor ESEM and bi-factor CFA versions, and; f) the reliability estimates (i.e., α, ω, etc.) of the ESEM should be acceptable [9, 12].

Under these conditions, flexible ESEM practices may enhance the findings of a wide range of modeling aims broadly used in psychiatry and mental health [7, 9]. These may involve the confirmation of predefined factor structures, the investigation of the interrelationships of different latent factors, measurement invariance procedures across different groups and over time, and even latent growth modeling [10]. Therefore, ESEM could be broadly applicable in the context of psychopathology/psychiatric scales employed for epidemiological, clinical intake assessment and intervention monitoring purposes [9].

## How can ESEM be operationalized?

Despite the ESEM theoretical background and utility having been articulated [10] and newer ESEM versions (e.g., set-ESEM; EWC [16]) developed, certain restrictions limit researcher implementation. Specifically, lack of flexibility in defining the model parameters, reporting rigidity, reproducibility of results, software accessibility and syntax/coding complexities reduce the adoption of its various versions (see 1a, 1b, 2, Set-ESEM and EWC variations described above [9]). Indeed, the ESEM 1a pathway appears to have been mostly applied, via the additional allocation of differential loadings for all non-primary items through the consideration of a close to 0 factor threshold in Mplus CFA procedures [8, 20]. The broader use of this ESEM pathway has been greatly supported by the ESEM code generator for Mplus introduced by de Beer and Van Zyl [20]. This allows less experienced Mplus users to automatically transform their multifactorial CFA models into their corresponding ESEM structures in order to proceed with testing [9]. While Mplus presents an excellent option for running these analyses, its limited accessibility (i.e., paid subscription) may hinder the broader ESEM adoption. Alternatively, a freely-accessible platform, such as RStudio, may present with greater flexibility and ease of accessing/editing syntax. Table 3 provides an overview of RStudio advantages.

**Table 3:** Comparison between Mplus and RStudio

Consideration	**Mplus**	**R / RStudio**
Flexibility in settings running models	Restricted to options available in Mplus	Flexible and customizable with a number of available packages to address various stages of modelling and user-led customization of the model run(s)
Flexibility in model reporting	Restricted to standardised output options	Flexible and customizable
Flexibility in results presentation for academic publication	Requires the results to be copied/extracted from Mplus, pasted and edited for the final document	Highly customizable with functionalities for preparation of submission ready output formats
Reproducibility of the results	Reduced due to locked-in Mplus environment	Reproducible with no restrictions, as model generation and reporting can be integrated in results
Syntax	The factor structure needs to be defined manually. A syntax generator is available separately	Factor structure can be either defined manually or can be automated with multi modelling functionalities and nested model runs
Cost	Mplus license is required	R and RStudio is free for academic purposes

In that context, the broader use of the ESEM 1b and the ESEM 2 pathways are feasible in R software via the currently openly available “esemComp” R package [19] and the ESEM/EFA-based code introduced by Revelle [18]. The esemComp operationalization of the ESEM pathway 1b appears to be more user-friendly and comparable to the Mplus ESEM calculation [19]. Nevertheless, its adoption is likely compromised by modeling challenges related to the multiple steps required (i.e., distinct EFA and ESEM steps, similar to EWC, except for the first step requiring EFA, instead of ESEM, to inform the loading starting points for the CFA at step 2). Additionally, the esemComp assumes that users can correctly identify factor-referent items, resulting in likely human error [19]. To address these limitations, the current tutorial provides an ESEM R code that merges EFA and ESEM-CFA modeling steps while automating the selection of factor-referent items. More importantly, the approach proposed in this tutorial produces similar (and potentially improved results) to those obtained via the Mplus alternative, as it enables varying calculation thresholds for all items. To demonstrate the implementation of the method, this tutorial will use the Strengths and Difficulties Questionnaire (SDQ [24]) given its questioned CFA and ESEM factor structure(s) in a series of earlier studies [8, 23, 25]).

## An ESEM tutorial example: The SDQ controversial factor structure

The SDQ is a popular mental health instrument used in several studies nationwide [26–28] to assess psychological strengths and difficulties for individuals aged between 2 and 17 [8, 29]. It includes 25 items distributed across five scales addressing Emotional Symptoms (ES), Conduct Problems (CP), Hyperactivity (Hy), Peer Problems (PP), and Prosocial Behavior (PB [30]). The same 25 items, with respondent-specific wording variations, can be completed by parents and teachers, as well as self-reported by the assessed child/adolescent [8, 30].

**Fig. 2:**
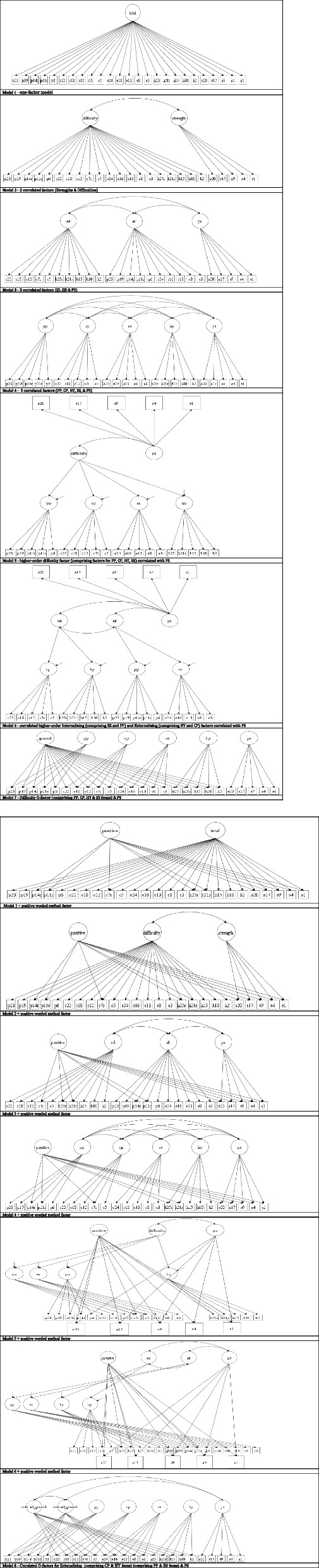
SDQ models proposed in previous literature

The SDQ structure has been challenged with different proposed factorial models across various national and age samples [8, 23, 29, 31, 32]. For example, working with Malaysian parental SDQ ratings for children 5–13 years, Gomez and Stavropoulos [29] demonstrated support for an oblique six-factor model involving aside of the five SDQ domains a positive construal factor comprising all the 10 SDQ positive worded items [29]. Furthermore, at least three studies have examined the potential fit of ESEM SDQ models. Firstly, Garrido and colleagues [25] analysed a Spanish-speaking population of 67,253 SDQ respondents (10–18 years) and found the five-factor CFA SDQ structure biased, with its respective ESEM version presenting a rather weak factorial structure. Secondly, Black et al. [23] investigated the SDQ responses of 30,842 UK students (11 to 15 years) and found the five-factor ESEM model valid for Year 7 and 9 students. Finally, Gomez and colleagues [8] examined the SDQ responses of 968 Greek-speaking adolescents (12–18 years) and supported an ESEM model with three factors entailing dysregulation, peer problems, and prosocial behavior, whilst also recommended further research (see supplementary Table 1 and Fig. 2 for more detailed information [29]). Furthermore, researchers have flagged only partial measurement invariance across different SDQ language versions, with the English versions of the instrument (such as the one used for the current tutorial) showing a particularly ill-fit [32]. Interestingly, the latter has been attributed to items simultaneously loading on several latent factors, reinforcing the need for ESEM testing [32]. The above prompted scholars to suggest that due to the SDQ multidimensionality and the close relationship between SDQ factors, the five-factor structure should be tested via ESEM [8].

## Aims of the present tutorial

Considering the potential ESEM benefits, deterrents, and recent recommendations for simplifying ESEM R procedures [9], this work aims to equip younger scholars with a tutorial implementation of ESEM pathway 1b via a newly introduced R code/automation. To achieve this, the contested SDQ factor structure will be used as an example [29]. For brevity and to avoid repetitions with existing literature regarding ESEM reporting guidelines [9]) and the optimum SDQ factor structure [8, 23, 25, 29, 30], only the conventional five-factor CFA and its corresponding ESEM will be examined here [9].

## ESEM tutorial: Methods, materials and procedure

A subset of pre-existing data (i.e., Growing Up in Australia: The Longitudinal Study of Australian Children [LSAC]) was used (https://dataverse.ada.edu.au/dataverse/lsac [33, 34]), including 3956 participants from cohort K (i.e. Kinder Cohort, approximately 5000 children between 4–5 years in 2003/2004) to the SQD. This questionnaire includes 25 items rated on a three-point Likert scale ranging from 0 to 2 (0 = “*not true*”, 1 = “*somewhat true*”, 2 = “*certainly true*”). Items are equally distributed across five proposed domains involving ES, CP, Hy, PP, and PB [26, 27]. A retrospective power analysis via the semPower R package indicated that a model with α = 0.05, *df* = 190, and N = 3836 would yield acceptable power (1-β = 0.99), being satisfied by the current sample size [35] (see Supplementary Fig. 1 for more details).

Ethics approval to use the archival LSAC data was granted by the Victoria University Human Research Ethics Committee on 10^th^ May 2022. The original data collectors obtained written and verbal consent from parents/guardians and, where appropriate, from the participants. All procedures performed involving human participants were in accordance with the 1964 Helsinki Declaration and its later amendments. Permission to access and utilize the dataset was provided by the National Centre for Longitudinal Data, Australian Government Department of Social Services on 14^th^ July 2021.

## ESEM Tutorial: A step-by-step ESEM guide via the R software using the SDQ five-factor structure example

The following section will use the ESEM package (https://cran.r-project.org/web//packages/esem/esem.pdf [36]) to demonstrate the expansion of the traditional five-factor SDQ CFA model with the inclusion of loading calculation thresholds derived via a) fixed rates approximating 0 (Mplus approach [20]) and b) prior ESEM embedded EFA procedures/loadings via the R software. Considering the latter, the full list of ESEM implementation R functions is provided in the GitHub html appendix, while the exact data used in the tutorial is also attached (data available from https://github.com/maria-pro/test/blob/master/ESEM/data/lsac.sav).

## 1a Pathway: ESEM based on fixed loading thresholds approximating 0 via Mplus

ESEM modeling can be performed through the expansion of traditional CFA with the inclusion of all non-primary factor-item loadings at a fixed rate approximating 0. Beer and van Zyl [20] have introduced a peer-reviewed Mplus ESEM generator online software for any chosen Mplus model defined (see http://www.surveyhost.co.za/esem/). Nonetheless, this section provides detailed instructions to fit an ESEM with zero approximation of non-primary item loadings using Mplus syntax.

**Fig. 3:**
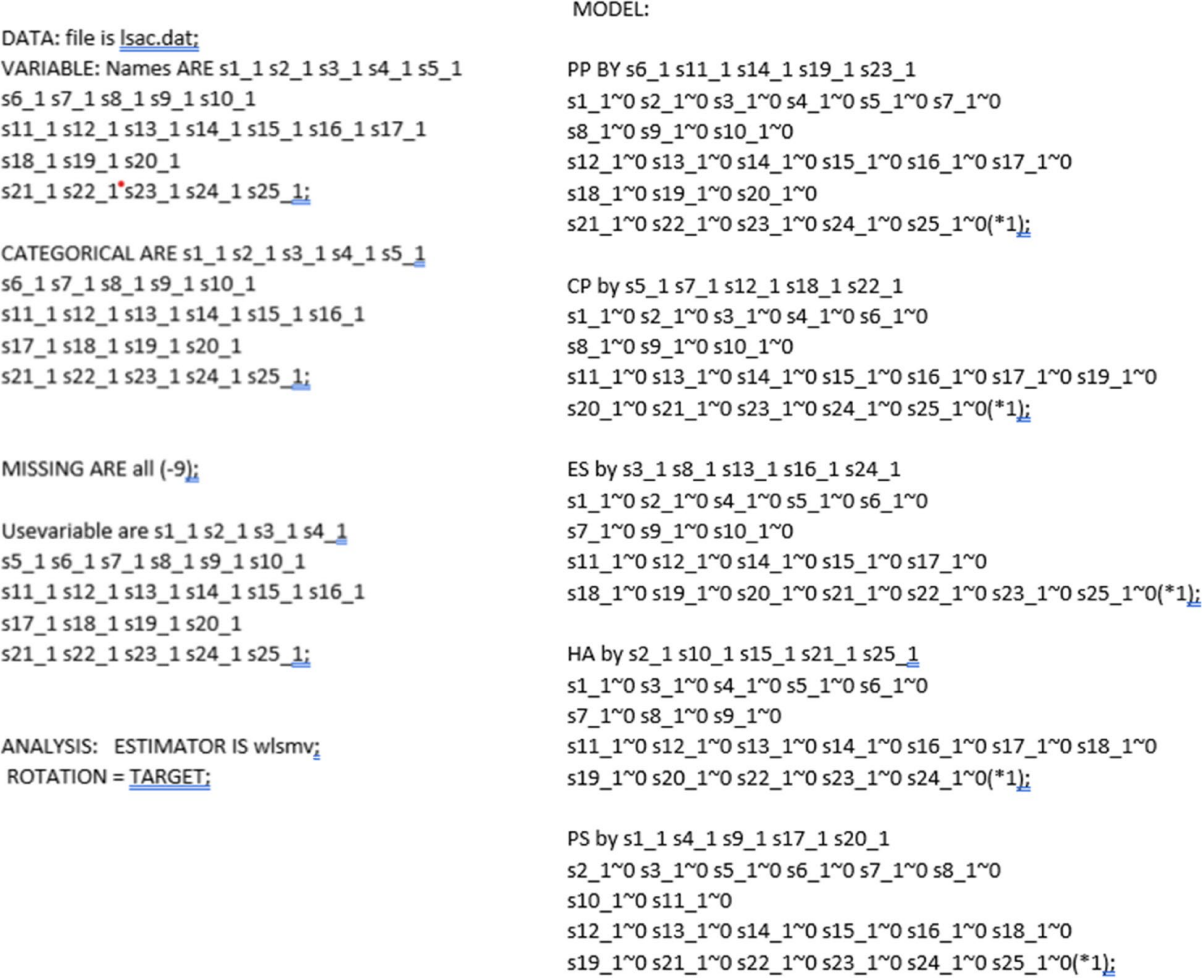
Defining the model in the Mplus environment

Firstly, fitting an ESEM model using Mplus requires loading the data (see *Loading the Data,* Table 4, Setup). Subsequently, the variables should be named (see *Naming the Variables,* Table 4, Setup) and their nature defined (see *Defining the nature of the variables,* Table 4, Setup). Researchers must identify the variables to be used for the analysis (see *Variables to be used*), define missing values (see *Defining missing values,* Table 4, Setup), the estimator (in this case, WLSMV), and the rotation*.* The model is then prepared to identify primary items loadings in each latent factor and non-primary items loadings constrained to approximately zero (~ 0; see *Model setup,* Table 4, Step 1; and Fig. 3). Subsequently, the model is tested, and results are produced (see *Testing the ESEM model,* Table 4, Step 2).

**Table 4:** Pathway 1a: ESEM based on fixed cross-loading thresholds approximating 0 – Mplus syntax

Procedure Steps	Aims	Mplus code included in the*.inp* syntax file	Translation
Setup	- Defining the analysis title - Loading the Data -Naming the variables -Defining the nature of the variables, if CATEGORICAL - Defining missing values - The variables to be used in the analyses are also required to be defined - The analysis’ features are then required to be defined	# chosen title follows the command *Title:* Title: SDQ ESEM 5 factor model for time 1 data # data to be analysed follows the command *DATA: File is* DATA: file is data.csv; # variable names are provided after the command *VARIABLE: Names ARE* VARIABLE: Names ARE s1_1 s2_1 s3_1 s4_1 s5_1 s6_1 s7_1 s8_1 s9_1 s10_1 s11_1 s12_1 s13_1 s14_1 s15_1 s16_1 s17_1 s18_1 s19_1 s20_1 s21_1 s22_1 s23_1 s24_1 s25_1; # Categorical variables are provided after the command *CATEGORICAL ARE:* CATEGORICAL ARE s1_1 s2_1 s3_1 s4_1 s5_1 s6_1 s7_1 s8_1 s9_1 s10_1 s11_1 s12_1 s13_1 s14_1 s15_1 s16_1 s17_1 s18_1 s19_1 s20_1 s21_1 s22_1 s23_1 s24_1 s25_1; # The character(s) defining missing values are provided after the command *MISSING ARE all:* MISSING ARE all (-9); # The variables to be used in the analyses are provided after the command *Usevariable are:* Usevariable are s1_1 s2_1 s3_1 s4_1 s5_1 s6_1 s7_1 s8_1 s9_1 s10_1 s11_1 s12_1 s13_1 s14_1 s15_1 s16_1 s17_1 s18_1 s19_1 s20_1 s21_1 s22_1 s23_1 s24_1 s25_1; # The analysis’ features are then selected. After the command *ANALYSIS*, the type of estimator and rotation are provided via the commands *ESTIMATOR IS* and *ROTATION* = respectively ANALYSIS: ESTIMATOR IS wlsmv; ROTATION = TARGET;	The initial Mplus setup involves: a) defining the title of analyses; b) loading the data to be used; c) naming the variables included in the data; d) identifying “categorical” variables within the data; e) providing the missing values’ identifier; g) identifying the specific data variavbles to be used in the analyses and; h) definining the analyses’ estimator and rotation type
Step 1	Model setup	# The analysis’ CFA model is defined after the command *MODEL:* The latent factors are on the left side followed by “*BY”* indicating the items allocated to them. All non-prmary items are followed by ~ *0*, which requests their loadings to be modelled when exceeding a level approximating 0 (this is the exploratory part of the analyses). The last item for each latent factor is fixed (*1) MODEL: PP BY s6_1 s11_1 s14_1 s19_1 s23_1 s1_1 ~ 0 s2_1 ~ 0 s3_1 ~ 0 s4_1 ~ 0 s5_1 ~ 0 s7_1 ~ 0 s8_1 ~ 0 s9_1 ~ 0 s10_1 ~ 0 s12_1 ~ 0 s13_1 ~ 0 s15_1 ~ 0 s16_1 ~ 0 s17_1 ~ 0 s18_1 ~ 0 s20_1 ~ 0 s21_1 ~ 0 s22_1 ~ 0 s24_1 ~ 0 s25_1 ~ 0(*1); CP BY s5_1 s7_1 s12_1 s18_1 s22_1 s1_1 ~ 0 s2_1 ~ 0 s3_1 ~ 0 s4_1 ~ 0 s6_1 ~ 0 s8_1 ~ 0 s9_1 ~ 0 s10_1 ~ 0 s11_1 ~ 0 s13_1 ~ 0 s14_1 ~ 0 s15_1 ~ 0 s16_1 ~ 0 s17_1 ~ 0 s19_1 ~ 0 s20_1 ~ 0 s21_1 ~ 0 s23_1 ~ 0 s24_1 ~ 0 s25_1 ~ 0(*1); ES BY s3_1 s8_1 s13_1 s16_1 s24_1 s1_1 ~ 0 s2_1 ~ 0 s4_1 ~ 0 s5_1 ~ 0 s6_1 ~ 0 s7_1 ~ 0 s9_1 ~ 0 s10_1 ~ 0 s11_1 ~ 0 s12_1 ~ 0 s14_1 ~ 0 s15_1 ~ 0 s17_1 ~ 0 s18_1 ~ 0 s19_1 ~ 0 s20_1 ~ 0 s21_1 ~ 0 s22_1 ~ 0 s23_1 ~ 0 s25_1 ~ 0(*1); HA BY s2_1 s10_1 s15_1 s21_1 s25_1 s1_1 ~ 0 s3_1 ~ 0 s4_1 ~ 0 s5_1 ~ 0 s6_1 ~ 0 s7_1 ~ 0 s8_1 ~ 0 s9_1 ~ 0 s11_1 ~ 0 s12_1 ~ 0 s13_1 ~ 0 s14_1 ~ 0 s16_1 ~ 0 s17_1 ~ 0 s18_1 ~ 0 s19_1 ~ 0 s20_1 ~ 0 s22_1 ~ 0 s23_1 ~ 0 s24_1 ~ 0(*1); PS BY s1_1 s4_1 s9_1 s17_1 s20_1 s2_1 ~ 0 s3_1 ~ 0 s5_1 ~ 0 s6_1 ~ 0 s7_1 ~ 0 s8_1 ~ 0 s10_1 ~ 0 s11_1 ~ 0 s12_1 ~ 0 s13_1 ~ 0 s14_1 ~ 0 s15_1 ~ 0 s16_1 ~ 0 s18_1 ~ 0 s19_1 ~ 0 s21_1 ~ 0 s22_1 ~ 0 s23_1 ~ 0 s24_1 ~ 0 s25_1 ~ 0(*1);	This approach firstly requires a traditional CFA structure, which allocates the primary indicators/items to their primary hypothesized latent factors. Factors are named on the left side of “by” and items are following on the right side. All non-primary items (i.e. crossloadings) are followed by ~ 0 to indicate approximate to 0 loadings to be calculated. For scaling purposes, the final item of each factor is followed by (*1)
Step 2	Testing the ESEM model	# The OUTPUT: command is followed by *standardized;* and *stdyx;* to request standardised outcomes for categorical covariates. *tech 4;* option is used to request estimated means, covariances, and correlations for the latent variables in the model. Finally *mod (10);* indicates the extraction of modification indices when the modification index for a parameter is greater than or equal to 10 OUTPUT: standardized;stdyx; tech4; mod(10);	This step produces the model results calculations

**Fig. 4:**
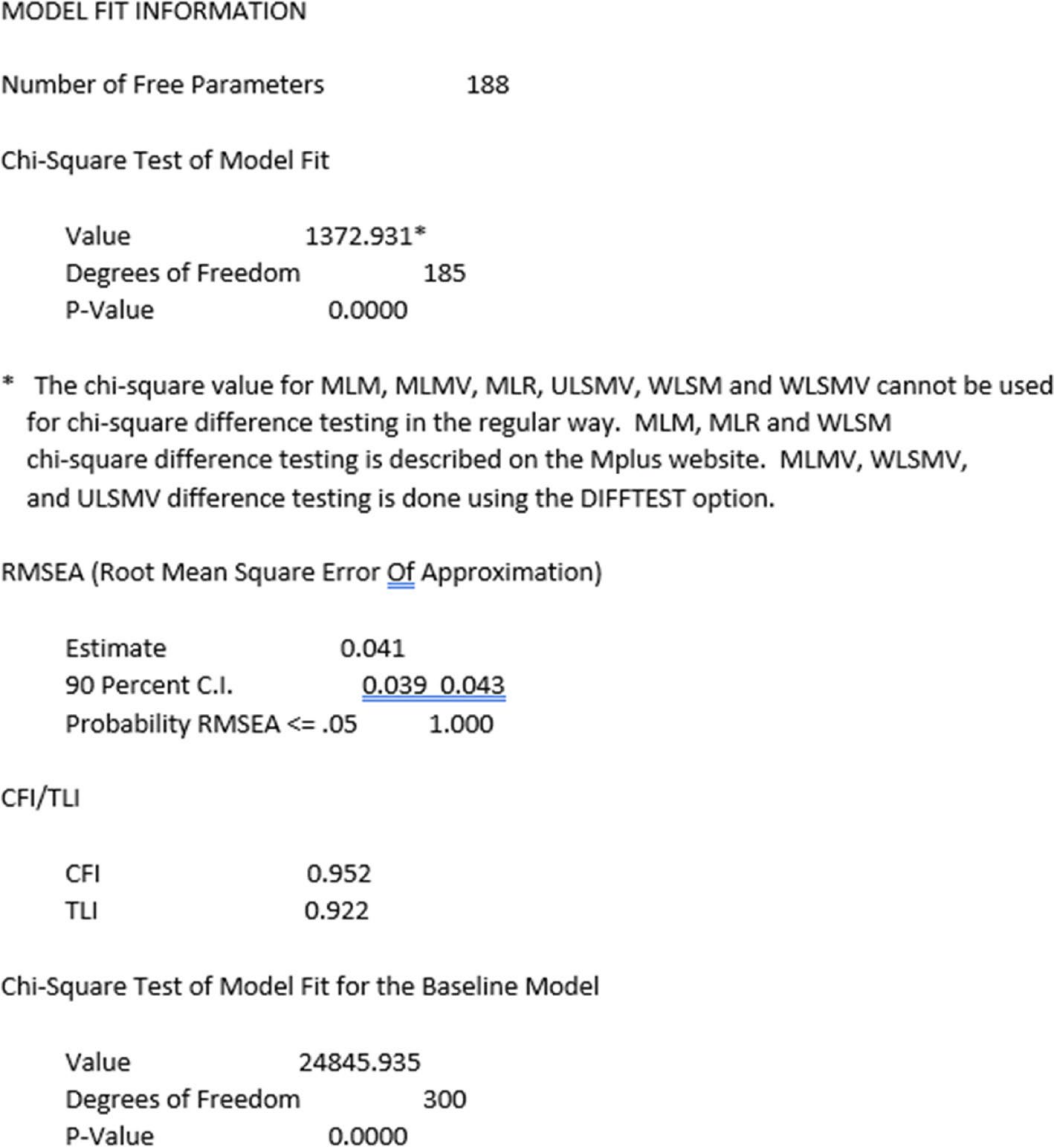
ESEM summary information using Mplus environment

Using these steps, a five-factor ESEM was fitted with all non-primary items approximating zero (~ 0). This model showed acceptable fit indices (χ^2^_[185]_ = 1372.931, *p* < 0.001, CFI = 0.952, TLI = 0.922, RMSEA = 0.041), most items loaded significantly on all five latent factors and latent factors covaried (see Fig. 4).

## 1b Pathway: ESEM based on EFA derived loading thresholds via RStudio

Alternatively, before conducting the ESEM procedure, an EFA is required to extract the factor loadings that will be used to expand the traditional CFA model (both processes are embedded within step 1 of the proposed R code). To achieve this, the EFA loadings and the specific factor referent items are automatically summarized as a structural unit, which directly informs the creation of the ESEM analysis. The ESEM model is then tested (Table 5, Step 2) and visualized (Table 5, Step 3). It is noted that Geomin rotation is, by default, embedded in step 1 (Table 5). If a researcher prefers target rotation for more theory-driven and less exploratory results, Step1 should be substituted by the Step1a alternative code (see Table 5). In either case, the end-product ESEM model behaves as a “conditional” CFA procedure, where factors are calculated based on all their primary and non-primary item loadings, provided these exceed their EFA varying levels. This improves modeling accuracy compared to 1a Pathway^2^.

**Table 5:** Pathway 1b: ESEM based on EFA extracted loading thresholds (as discussed in [16])

Procedure Steps	Aims	R code	Translation
Setup	- Installing R packages -Loading R packages for further data exploration and—Enabling data preparation, including analysis of outliers and missing values—Enabling the selection of the estimation method and rotation appropriate for the data and study aims - Loading the dataset	#Installation is done only once before the packages can be used in RStudio install.packages("tidyverse") install.packages("psych") install.packages("lavaan") install.packages("GPArotation") install.packages("semPlot") remotes::install_github("maria-pro/esem", build_vignettes = TRUE) #Loading packages is required at the start of the analysis to get access to functions, and in the case of this tutorial, datasets available in packages library(esem) library(tidyverse) library(lavaan) library(semPlot) library(psych) #Loading the demonstration data sdq_lsac < -sdq_lsac describe(sdq_lsac)	The initial setup includes installing packages that will be used for analyses, using *install.packages()* function for packages from CRAN^1^ “tidyverse”, “psych”, “lavaan”, “ semPlot” and remotes::install_github() function for non-CRAN packages available from github^2^ (select option 1 upon installation of esem package). Installation is done one time and is not required every time the packages are loaded for use Loading of packages using *library()* function is required first, to make functions and datasets in packages of interest available for use in RStudio The psych package is used for the EFA procedure The lavaan and semPlot packages are used for the CFA related steps The esem package/ R code was developed specifically for the current paper to simplify and demonstrate ESEM free of cost procedures. It includes the dataset, as well as relevant ESEM automated functions employed in the demonstration example Data preparation can be achieved using the tidyverse package, which targets data exploration and visualization. The dataset used in this tutorial has already been cleaned and the details of the pre-processing are available in the supplementary material via the github repository *sdq_lsac* is the in-built dataset provided in the *esem* package. *sdq_lsac* loads the dataset in a variable called *sdq_lsac* (the name of the variable can be changed per user preference) *describe()* provides basic statistics for the *sdq_lsac* dataset
Step 1	Conduct full ESEM via embedding EFA, Geomin rotation, derived cross-loadings in CFA	esem_results < -esem_c(data = sdq_lsac, nfactors = 5, fm = 'ML', rotate = "geominT", scores = "regression", residuals = TRUE, Target = NULL, missing = TRUE, mimic = c("MPlus"), std.lv = TRUE, ordered = TRUE)	The *esem_c()* function estimates and reports ESEM results. The results are then saved in esem_results object The following arguments are used: - the dataset to be used *data* = *sdq_lsac*, alteratively, a correlation or covariance matrix can be provided - the number of factors *nfactors* = *5* (based on the classic 5-factor SDQ approach in literature) - the evaluation is done using the ML algorithm, *fm* = *'ml'*. The alternative algorithms are available, including minimum residual (*minres*, i.e. *ols* or *uls*), principal axes, alpha factoring, weighted least squares and minimum rank. The full list of algorithms is provided at https://www.rdocumentation.org/packages/psych/versions/2.2.3/topics/fa - the rotation method *rotate* = *"geominT"*. The full list of available rotations is accessible at https://www.rdocumentation.org/packages/psych/versions/2.2.3/topics/fa - factor scores are estimated using regression via *scores* = *"regression"*. Alternative approaches are available at at https://www.rdocumentation.org/packages/psych/versions/2.2.3/topics/fa - *residuals* = *TRUE* requests the residual matrix to be generated and presented *- Target* = *Null,* to indicate to target item/factor rotation - the dataset used in this tutorial (*sdq_lsac)* has no missing values, but for demonstration purposes the argument *missing* = *TRUE* is used – it allows to impute missing values, in case these occur -*mimic* = *c(“Mplus”)* indicates a calculation that follows the Mplus procedure (pathway 1a), with the exception that item loading thresholds for the ESEM modelling stage are defined by the automated EFA results *- std.lv* = *TRUE* indicates that standardised values are produced at the modelling stage -ordered = True - the default confidence intervals for RMSEA is used with *alpha* = *.1* - the default probability values are used for confidence intervals; however they can be adjusted by specifying *p* and the value. The default is *p* = *.05* For more options on running the *esem_efa()* function please see https://www.rdocumentation.org/packages/psych/versions/2.2.5/topics/fa Please ignore the *“Loading required namespace: GPArotation”* message received, as such functions are already addressed by the packages retrieved The alternative solution is to run EFA with Target rotation. This option is explained in the alternative Step1a below
Step 1a	Conduct EFA to calculate EFA derived cross-loadings with Target rotation	main_loadings_list <—list( pp = c("s6_1", "s11_1R", "s14_1R", "s19_1", "s23_1"), cp = c("s5_1", "s7_1R", "s12_1", "s18_1", "s22_1"), es = c("s3_1", "s8_1", "s13_1", "s16_1", "s24_1"), ha = c("s2_1","s10_1","s15_1","s21_1R","s25_1R"), ps = c("s1_1","s4_1","s9_1","s17_1","s20_1")) target < -make_target(data = sdq_lsac, keys = main_loadings_list) esem_results < -esem_c(data = sdq_lsac, nfactors = 5, fm = 'ML', rotate = 'TARGET', scores = "regression", residuals = TRUE, Target = target, missing = TRUE, mimic = c("Mplus"), std.lv = TRUE, ordered = TRUE)	-For target rotation, there needs to be a target loadings’ matrix supplied to the EFA analysis -To make the *target* matrix object, a list of main loadings (*main_loading_list*) is created using the *list()* function and supplied to the *make_target()* function -The *esem_efa* function, then explores the *data*, here defined as *sdq_lsac* -. The number of factors selected needs to correspond with the number of factors defined in the *main_loadings_list* object. For this example this is 5 The *esem_efa()* function is used with *rotate* = *“TargetQ"* and target matrix is provided as *Target*. All other arguments remain the same as in Step1
Step 2	Inspect the ESEM model	summary(esem_results, fit.measures = TRUE, standardized = TRUE, ci = TRUE)	To review the results the *summary()* function is used with: -*fit.measures* = *TRUE*. This calculates the goodness of fit parameters to assess model fit of the *esem-results* object defined in either Step1 or Step1a The argument Standardized = TRUE provides two columns reporting (i) standardized parameters when only the latent variable is standardized (std.lv), and (ii) standardized parameters when both observed and latent variables are standardized (std.all) For more options on running esem_cfa() function please see https://www.rdocumentation.org/packages/lavaan/versions/0.5-9/topics/cfa
Step 3	Visualizing ESEM Model	semPaths(esem_results,whatLabels = "std",layout = "tree")	The *semPaths ()* function plots the model and allows to customise its visualization with the following arguments: **-** *esem_fit* as the fitted model, created in step 4 **-** *whatLabels* = *”std”* to produce standardized path coefficients - *layout* = *”tree”* to produce a tree-like disposition of elements in the plot

**Fig. 5:**
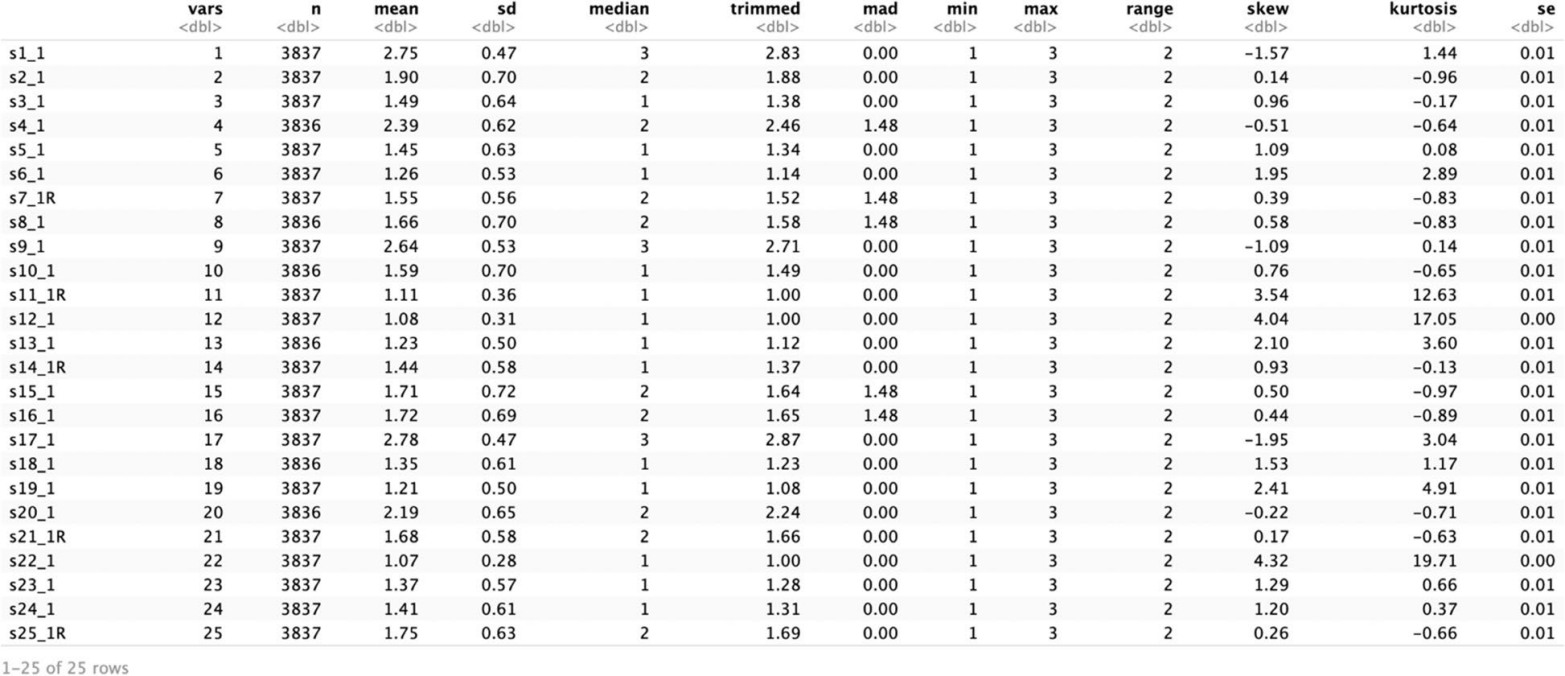
Descriptive statistics generated in R Studio

Estimating an ESEM model with R Studio requires the installation of ‘ESEM’, a dedicated package containing specific functions to ease this process. The package can be installed using *remotes::install_github("maria-pro/esem", build_vignettes* = *TRUE)* and loaded using *library(esem)* (Table 5, setup). Other packages are required to execute this example (i.e., tidyverse, psych, lavaan, GPArotation, and semPlot; see Table 5, setup [37–40]. The line *sdq* <—*sdq_lsac* loads the dataset used for this demonstration. A table with descriptives statistics can be obtained with the function *describe(sdq_lsac)* (see Fig. 5).

**Fig. 6:**
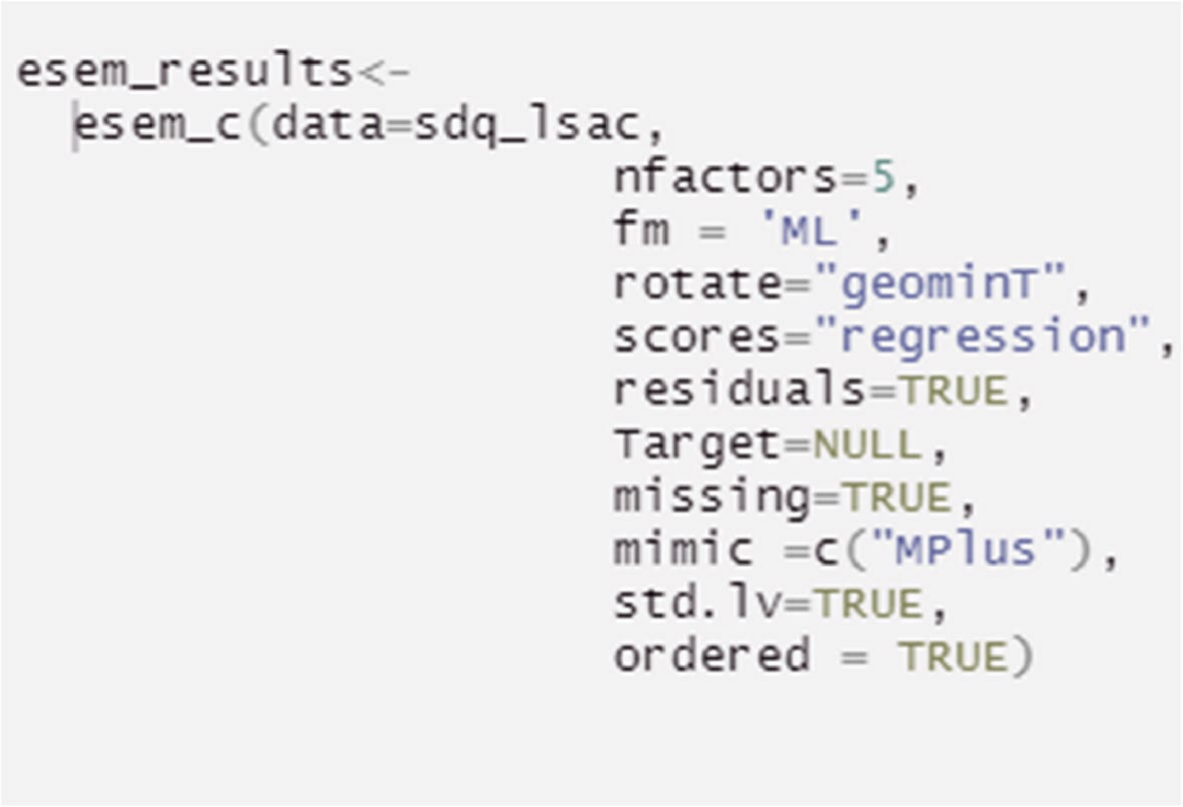
Fitting an ESEM with Geomin-rotation in R Studio

Subsequently, two approaches can be used to fit an ESEM model: (a) a Geomin rotation and (b) a targeted rotation. A Geomin rotation involves an exploratory approach (much like EFA), where the researcher can set the desired number of latent factors while allowing the algorithm to identify the main loading items on each latent factor (Table 5, Step 1; and Fig. 6).

**Fig. 7:**
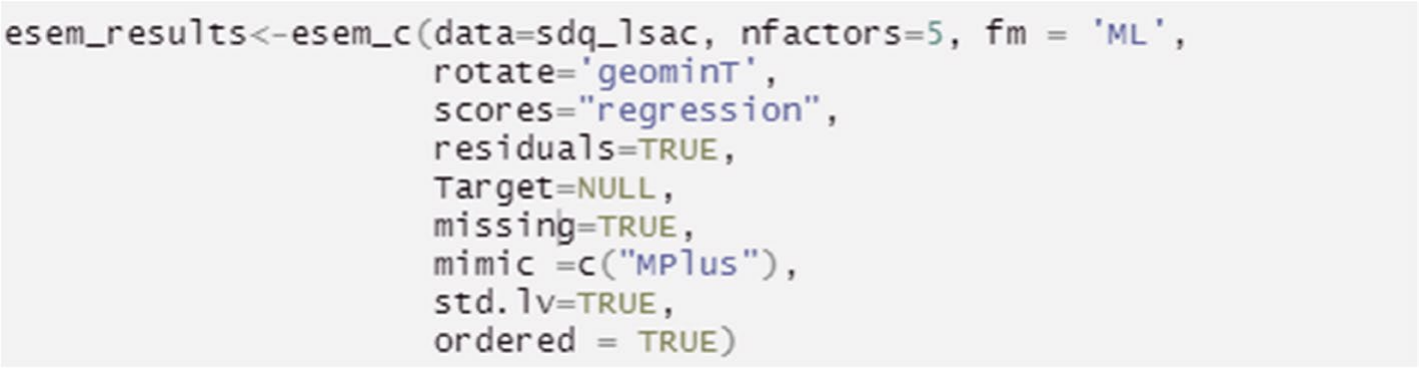
Fitting an ESEM with targeted rotation in R Studio

**Fig. 8:**
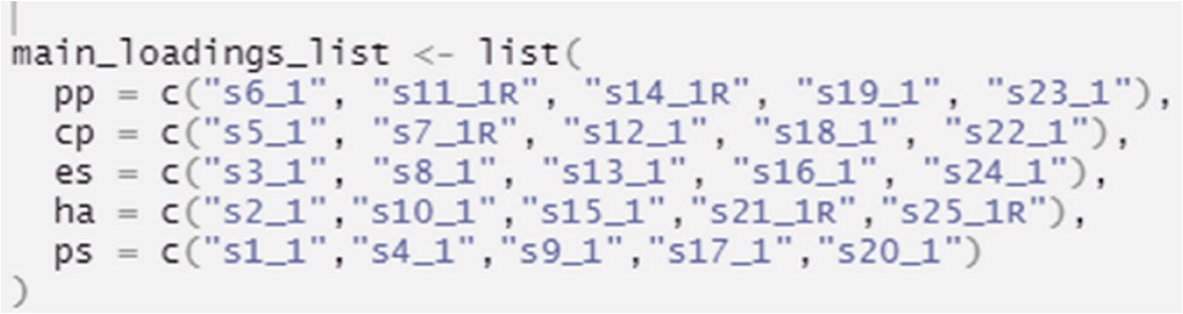
Fitting an ESEM with targeted rotation in R Studio

Alternatively, a targeted rotation involves creating a list object (*main_loadings_list*; Table 5, Step 1a; and Fig. 7), reproducing a desired factorial structure where the researcher predetermines the main loading items in each factor Fig 8.

**Fig. 9:**
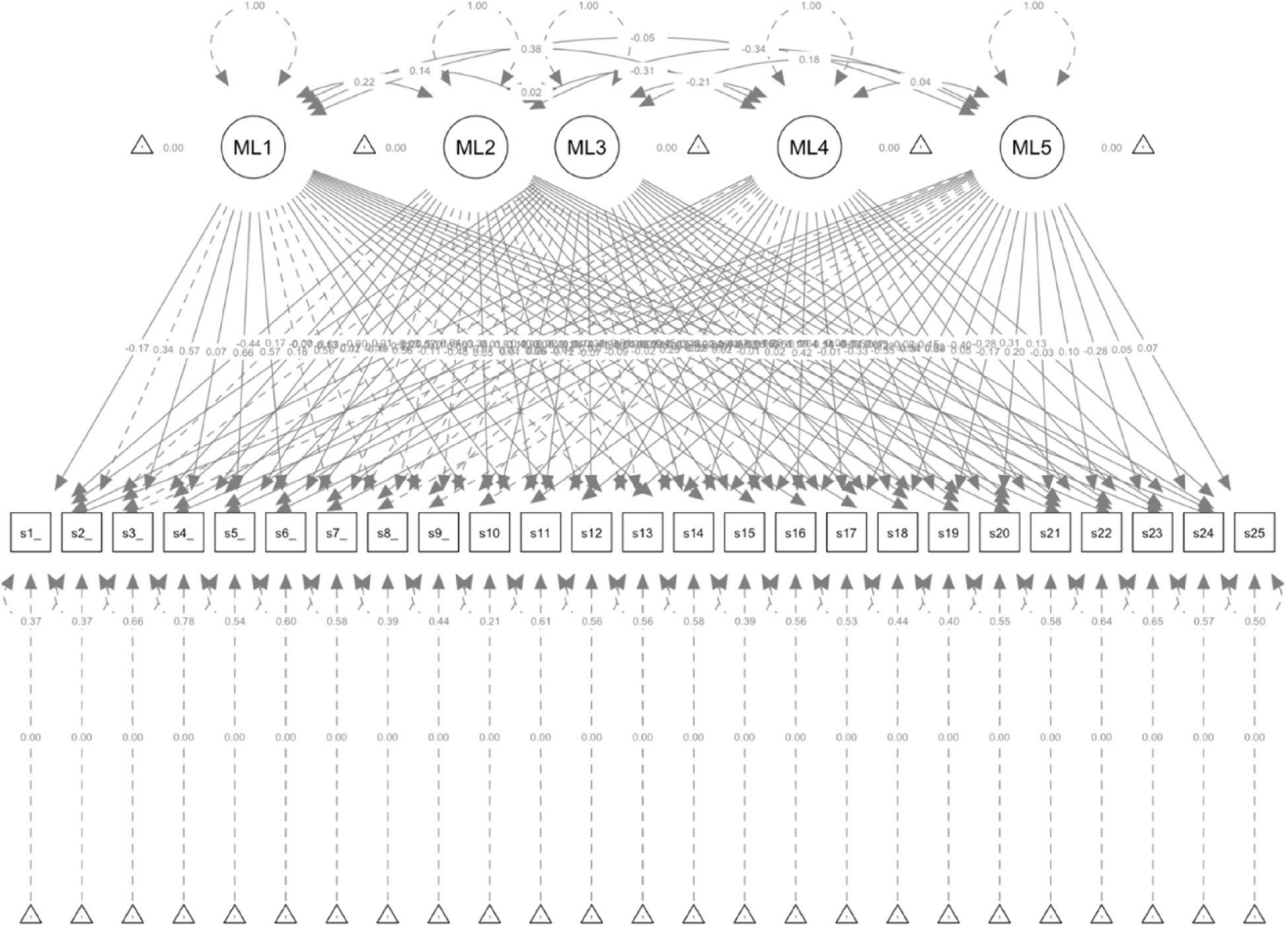
ESEM diagram

The function *make_target* enables the estimation of targeted loadings anchoring the lowest item-loading from each latent factor, and the function *esem_c* uses the target loading to fit an ESEM (Table 5, Step 1a). Finally, the results can be inspected using the function *summary* (Table 5, Step 2), and the factorial structure can be plotted using *semPaths* (Table 5, Step 3; and Fig. 9).

**Fig. 10:**
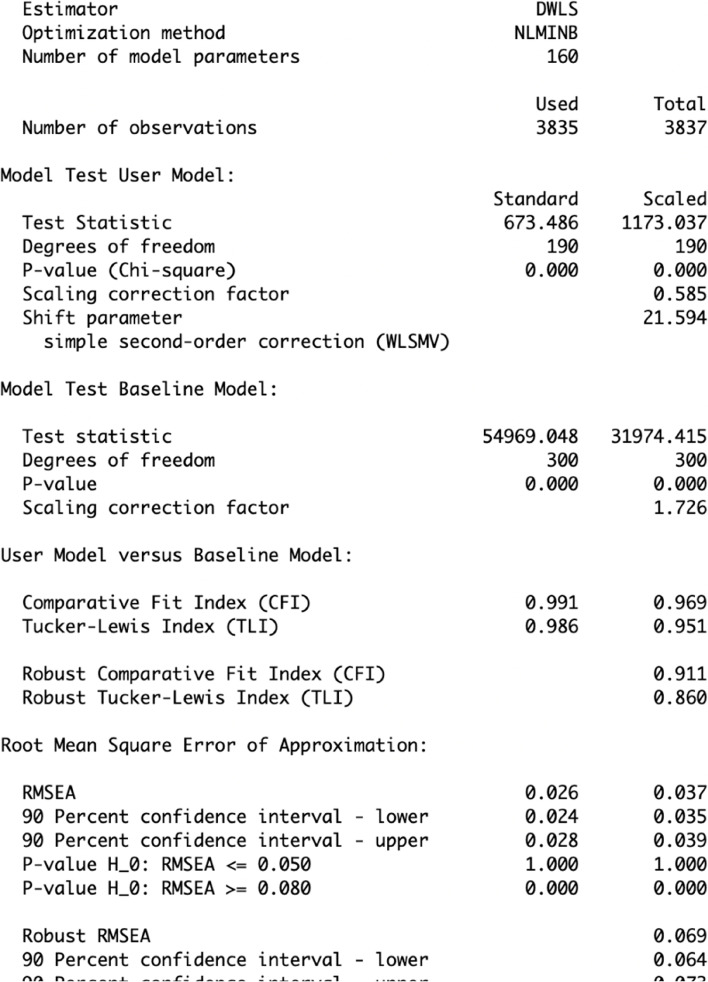
ESEM model results using pathway 1b step 1a

Using the steps described above, two five-factor ESEM were fitted, including Geomin and targeted rotation. Both models showed similarly acceptable fit indices (target rotation: χ^2^_[190]_ = 715.708, *p* < 0.001, CFI = 0.990, TLI = 0.985, RMSEA = 0.027, SRMR = 0.035; and Geomin rotation: χ^2^_[190]_ = 673.476, *p* < 0.001, CFI = 0.991, TLI = 0.986, RMSEA = 0.026, SRMR = 0.035; see Fig. 10). In both models, most items loaded significantly on all five latent factors, and most latent factors showed low covariance (see https://vas08011980.github.io/ESEM1b/ESEM1ba.html). A Satorra-Bentler chi-squared scaled difference test (SBS Δχ^2^) indicated no significant differences between both models (*p* = 0.99).

**Fig. 11:**
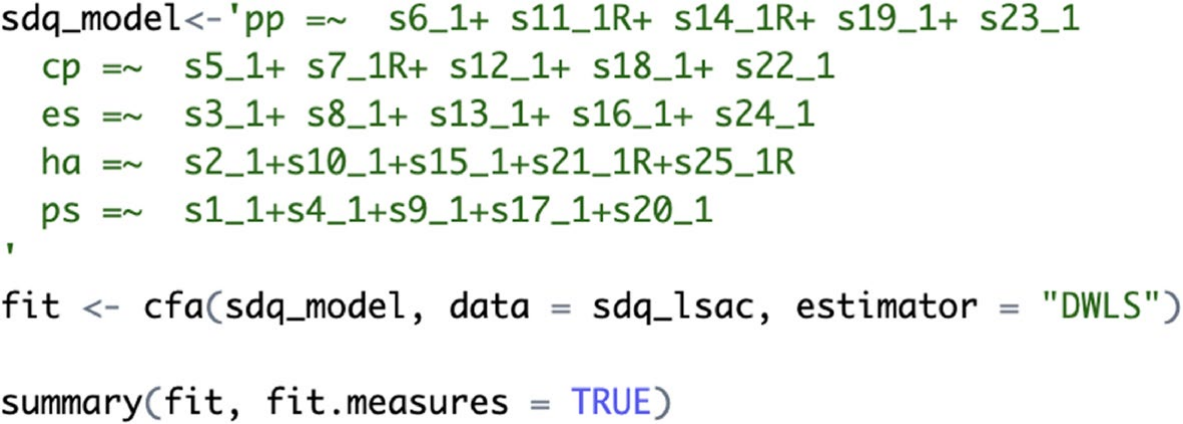
Running a CFA model in RStudio using the ‘lavaan package [39]

**Fig. 12:**
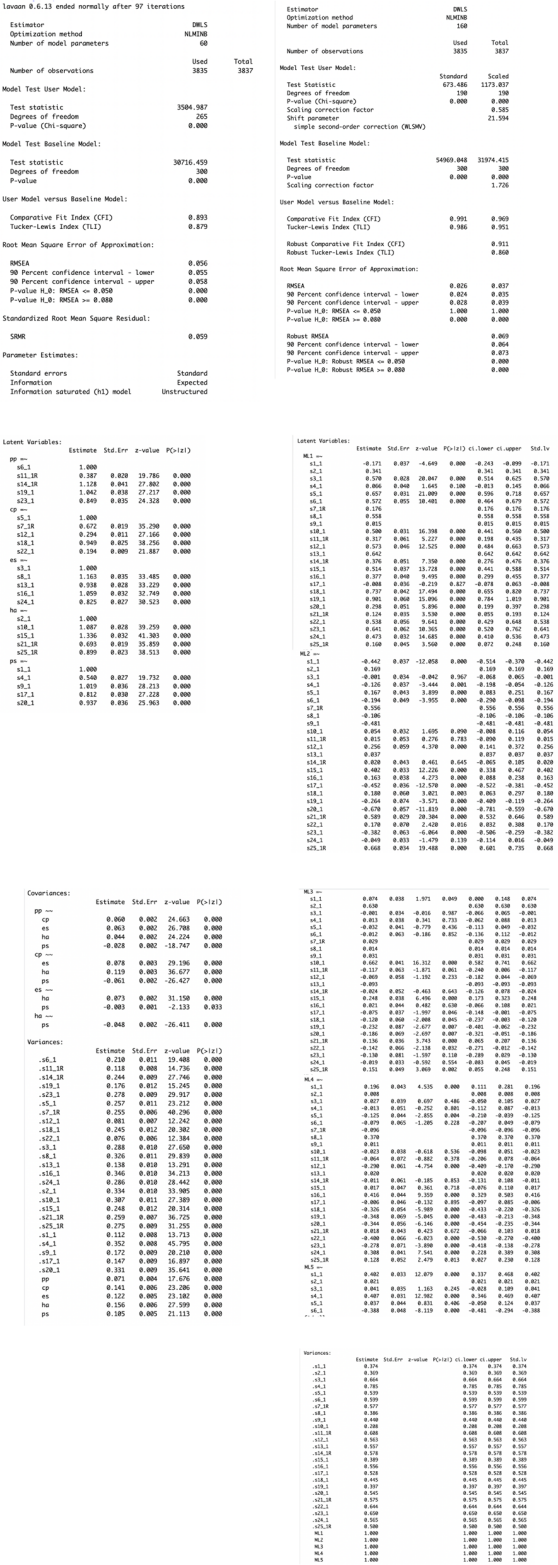
Comparing CFA and ESEM (with Geomin rotation, pathway 1b)

Considering the non-significant, albeit slightly improved fit of the ESEM model using a Geomin rotation (pathway 1b), the model was compared to its respective CFA model. The CFA analysis was conducted in R using `lavaan` package (see Fig. 11 [39]). The CFA model is specified as `sdq_model` and the analysis is conducted using *cfa()* function that specifies `DWLS` estimator. The results are presented below as R output for demonstration purposes (Fig. 12).

**Fig. 13:**
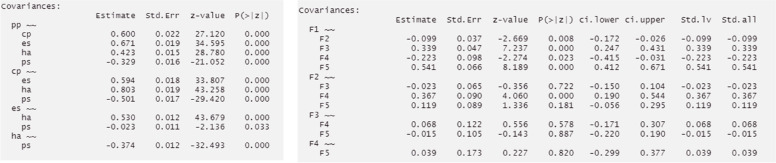
Comparing factor correlations between CFA and ESEM with Geomin rotation

The CFA model showed marginally acceptable fit indices (CFA: χ^2^_[265]_ = 3504.987, *p* < 0.001, CFI = 0.893, TLI = 0.879, RMSEA = 0.056, SRMR = 0.059). Moreover, a Satorra-Bentler chi-squared scaled difference test (SBS Δχ^2^) indicated that the ESEM with Geomin rotation showed a significantly better fit than its CFA counterpart (Δχ^2^_[75]_ = 265.850, *p* < 0.001). Most factor correlations were not significant in the ESEM model, while most were significant in the CFA model (see Fig. 13). The presence of item cross-loadings suggests that free estimation of non-primary items should be enabled; however, this process may obtain over-identified models capable of converging on a given solution [9, 12].

## Discussion

This tutorial addressed past recommendations to simplify and facilitate ESEM implementations [9]. To address this aim, it first provided an ESEM theoretical overview while emphasizing the comparison between ESEM and traditional EFA and CFA methods [12]. Secondly, different ESEM pathways and hybrid ESEM methodologies, including Set-ESEM and EWC were explored in relation to their potential estimator and rotation selection features [7, 9, 10, 12]. Thirdly, ESEM strengths, limitations, conditions and utility were briefly illustrated, and available ESEM operationalization procedures via the Mplus and the R Software were highlighted (alongside their advantages and disadvantages [9, 18–20]). Fourthly, the SDQ factor structure debate was succinctly explained to allow the use of the scale as an example for the current tutorial. Material and methods secured from the LSAC data were additionally described prior to the analyses [33]. To avoid repeating past literature, the SDQ five-factor CFA and its corresponding ESEM were emphasized in the context of the “Data Analysis and Reporting Phase” of the ESEM guidelines [8, 9, 23, 25, 29, 30]. Within this context, the present tutorial comparatively provided relevant R ESEM pathway 1b and Mplus syntax via a step-by-step guide, aiming to help young researchers implement this type of modeling.

Accordingly, the tutorial analyses demonstrated two different approaches to conduct ESEM via the Mplus and R software. These entail a) the inclusion of non-primary loading (i.e. cross-loadings) calculation thresholds via fixed rates approximating 0 (pathway 1a) and via ESEM embedded EFA procedures (pathway 1b). Pathway 1a shows ESEM analysis with the scale’s factor structure evaluated in a core confirmatory manner [20]. Alternatively, pathway 1b demonstrates ESEM embedding a prior CFA, EFA step to inform non-primary loadings calculation starting points [18]. Although similar to EWC, pathway 1b adopts an EFA and not an ESEM as an initial procedure, thus potentially being more methodologically rigorous than other ESEM modeling calculations [7]. The presentation of the results followed the suggested guidelines by van Zyl and ten Klooster [9] in the context of the required data analysis and reporting phase. In particular, the goodness-of-fit indices and measurement quality indicators are reported and benchmarked. The presentation of results is completed for both pathways to allow comparability of the approaches.

Comparison of global fit for all models was based on their CFI, TLI and RMSEA values and showed good fit with most items loading significantly on all five factors and the factors covarying, similar to past SDQ studies [15, 29]. Interestingly, the analysis additionally confirmed issues with the SDQ instrument, as items showed lower than 0.6 loadings on their designated factors aligning with past evidence [8, 23, 25, 29, 30]. The conducted ESEM was finally compared with its corresponding SDQ five factor CFA model [12]. Following suggestions by Morin et al. [12], the ESEM models are expected to show better data-model fit than CFA options except for smaller factor correlations in ESEM models, when compared to traditional CFA models. Thus, ESEM pathway 1b analysis was expected to show lower factor correlations (as it is a non-bifactor model). The cross-loadings in the ESEM model were envisaged to be below 0.5 and the estimated latent factors were also expected to show strong loadings matching expectations. Large cross-loadings in ESEM generally indicate the existence of a global factor and may present a case for bifactor ESEM [8, 23, 25, 29, 30].

Considering the comparison between ESEM pathway 1a (i.e., MPlus facilitated ESEM where the items’ cross-loadings with all their non-primary factors are uniformly set to approximate 0), and pathway 1b (where items’ cross-loadings with their non-primary allocated factors are calculated based on their EFA-derived thresholds), pathway 1b showed better fit. Specifically, whilst both procedures demonstrated sufficient fit, pathway 1b, as facilitated via the current proposed code showed lower chi-square, RMSEA and SRMR and higher CFI and TLI either with Target (i.e. χ2[190] = 715.708, p < 0.001, CFI = 0.990, TLI = 0.985, RMSEA = 0.027, SRMR = 0.035) or Geomin rotation (i.e. χ2[190] = 673.476, *p* < 0.001, CFI = 0.991, TLI = 0.986, RMSEA = 0.026, SRMR = 0.035) compared to pathway 1a facilitated via MPlus (χ2[185] = 1372.931, *p* < 0.001, CFI = 0.952, TLI = 0.922, RMSEA = 0.041). These results suggest that item-specific treatment, in the context of ESEM, may result in better fit indices. As this process is not available via MPlus, the present code is an attractive alternative for prospective ESEM users.

## Conclusions, implications, limitations & further research

Overall, considering the different ESEM calculation options applied, useful conclusions may be indicated. As such, one could support that ESEM calculation based on: a) the inclusion of fixed non-primary items thresholds approximating 0 (Pathway 1a); and b) EFA extracted loading thresholds (Pathway 1b), while similar, they are yet, to an extent, different. The second option allows EFA variability in primary item loadings to be also considered, while non-primary item loadings are differentially treated, according to their EFA performance. The first option initially distinguishes primary and non-primary item treatment, with no loading calculation thresholds included for primary items. Secondly, it uniformly addresses all non-primary items via the inclusion of the same approximating to 0 loading threshold. The comparison of the performance of the two alternatives in the context of this tutorial shows relatively improved fit, for pathway 1b (see GitHub html appendix). Overall, from a theoretical perspective, variability in the performance of even primary items is expected, and thus one would suggest that ESEM calculation based on EFA derived loading thresholds allows more reflective/accurate modeling.

As ESEM practices may enhance the usage of multidimensional questionnaires, the technique demonstrated in this tutorial has relevance for the critical appraisal of commonly used measures of human behavior, as well as behaviors with diagnosable psychopathology/psychiatric features (i.e., multifaceted mood, psychotic, and developmental disorders; e. g. bipolar; schizoaffective; attention deficit and hyperactivity; [41]). In addition, the present technique/code combines four significant strengths, as it: a) employs a publicly accessible software; b) provides an adaptable and easy to follow (even for less R-relevant users) ESEM code; c) utilizes a ‘real-world’ measure with a debatable factor structure/interpretation; and d) emphasizes on publicly accessible nationwide demonstration dataset. Overall, while ESEM conducted in both Mplus and ESEM presents valuable guidance for researchers, in regard to the SDQ instrument testing itself, the outcome requires further examination with the use of alternative ESEM/CFA models.

Nevertheless, such methodological strengths need to be evaluated in the context of a researcher’s required familiarity with the R software usage, as well as potentially significant ESEM limitations [9, 12]. These may involve occasional lack of parsimony and/or confusing factors, over-fitting risk, as well as the recommended cautiousness with the calculation of higher-order factors, partial invariance, mediation employed cross-loadings, multi-level, latent class and latent growth curve modeling [6, 9, 23]. In that line, and despite available ESEM literature converging to the usage of traditional CFA fit thresholds [42], the potential application of optimal ESEM cut-offs may need to be examined. Thus, we have now expanded our future research recommendations to address potentially ESEM-specific model fit thresholds Overall, ESEM modeling should not be perceived as an entirely positive procedure and/or without takings into consideration its limitations and specific uses (despite being able to solve many problems [7, 9, 12, 23].

*Note 1:* In Set-ESEM, the calculation of cross-loadings is enabled between predefined sets of factors, while they are prohibited to expand to different factor sets [6].

*Note 2:* Prior to the ESEM, data pre-processing including missing values analysis, outliers and distributional assumptions were addressed.

## Supplementary Information

**Additional file 1.** 

## Data Availability

The data of the current study is available on the following link https://github.com/maria-pro/esem. The package is available here https://cran.r-project.org/web//packages/esem/esem.pdf, and practical examples to reproduce analyses presented in the manuscript are available on the following links: step 1ba https://vas08011980.github.io/ESEM1b/ESEM1ba.html, and step ESEM 1b step 1 https://vas08011980.github.io/ESEM1b/ESEM1bpage.html
