# Supplementary material for: Exploratory structural equation modeling: a streamlined step by step approach using the R Project software
Source: BMC Psychiatry. 2023 Jul 28;23:546. doi: 10.1186/s12888-023-05028-9 (PMC10375619; doi:10.1186/s12888-023-05028-9)
Supplement: Supplementary file 1 — Additional file 1. [file 12888_2023_5028_MOESM1_ESM.docx]

Supplementary Table 1.

| SDQ Syndromes | Items Allocated |
| --- | --- |
| Peer problems (PP) | 6, 11, 14, 19, 23, |
| Conduct problems (CP) | 5, 7, 12, 18, 22 |
| Emotional Symptoms (ES) | 3, 8, 13, 16, 24 |
| Hyperactivity/inattention (HA) | 2, 10, 15, 21, 25 |
| Prosocial (PS) | 1, 4, 9, 17, 20 (all items worded positively) |

| **Models are those shown in Figure 1** |  |  |
| --- | --- | --- |
| **Model 1 to 6 are all first order factor models** | | |
| Model 1:one factor model |  | All 25 items on a single factor |
| Model 2: two-factor model | F1-Difficulties  PS | ES (3, 8, 13, 16, 24)  CP (5, 7, 12, 18, 22)  HA(2, 10, 15, 21, 25)  PP (6, 11, 14, 19, 23)  1, 4, 9, 17, 20 |
| Model 3: 3-correlated factor model | F1 -internalizing disorders(ID),  F2 - externalizing disorders(ED),  F3 - PS | ES (3, 8, 13, 16, 24) & PP (6, 11, 14, 19, 23) items  CP (5, 7, 12, 18, 22) and HA (2, 10, 15, 21, 25) items  1, 4, 9, 17, 20 |
| Model 4:Dickey & Blumberg (2004) 3-factor model (note item 22 not included in the model) | F1- internalizing disorders(ID),  F2- externalizing disorders(ED),  F3- PS | 3, 6, 8, 13, 16, 19, 23, 24  2, 5, 10, 12, 15, 18, 21, 25,7  PS (1, 4, 9, 17, 20) items, 7 |
| Model 5: 5-correlated factor model (PP, CP, ES, HA, PS) | F1- PP,  F2- CP,  F3- ES,  F4- HA,  F5- PS | 6, 11, 14, 19, 23,  5, 7, 12, 18, 22  3, 8, 13, 16, 24  2, 10, 15, 21, 25  1, 4, 9, 17, 20 |
| Model 6: 5 correlated factors with positive construal factor | F1- PP,  F2- CP,  F3- ES,  F4 -HA,  F5- PS  F6- positive construal factor | 6, 11, 14, 19, 23,  5, 7, 12, 18, 22  3, 8, 13, 16, 24  2, 10, 15, 21, 25  1, 4, 9, 17, 20  PS (1, 4, 9, 17, 20) and the 5 reversed key items (7, 11, 14, 21, 25). |
| Model 7: **Higher-order factor** model with Difficulty correlating with  the primary factor for PS | Difficulty HO factor with primary factors for  PP (6, 11, 14, 19, 23),  CP (5, 7, 12, 18, 22),  HA (2, 10, 15, 21, 25), and  ES (3, 8, 13, 16, 24  PS | 1, 4, 9, 17, 20 |
| **Higher order factor model** | | |
| Model 8: a **higher order** factor  (All factors correlated) | HO F1: Iinternalizing Disorders (ID) with primary factors for  ES and PP  HO F2: Externalizing disorders (ED) with primary factors for HY and CP)  Primary factor: PS |  |
| **Model 9 to 11 are Bifactor Models** | | |
| Model 9: bifactor orthogonal first-order factor model with a general factor and five specific factors | G-factor  ES specific  PP specific  HA specific  CP specific  PS specific | All items  ES items  PP items  HA items  CP items  PS items |
| Model 10: Caci et al. (2015)’s bifactor model first version  Difficulty and PS correlated. | G-Difficulty  ES specific  PP specific  HA specific  CP specific  PS primary factor | ES, PP, HA, CP items  ES items  PP items  HA items  CP items  All PS items |
| Model 11: Caci et al. (2015)’s bifactor model second version – two general factors  F, F2 and F3 are correlated. | F1. General factor ED comprising all CP and HY  items)  F2. General factor ID (comprising all PP and ES items)  F3. Primary for PS  Specific CP  Specific HY  Specific PP  Specific ES |  |
| **Model 12 was proposed by Stokes (based on Malaysian sample)** | | |
| Model 12: A three-factor model  This model can be ignored in your study | F1 - reactive and anxious behaviors  F2 - positive behaviors  F3 - anxieties, attention,  and obedience | 3,  5 8, 10, 13, 15, 16, and 24  1, 4,  7, 9 14, 17, and 20  1, 7, 16, 16, 21, and 25 |

| 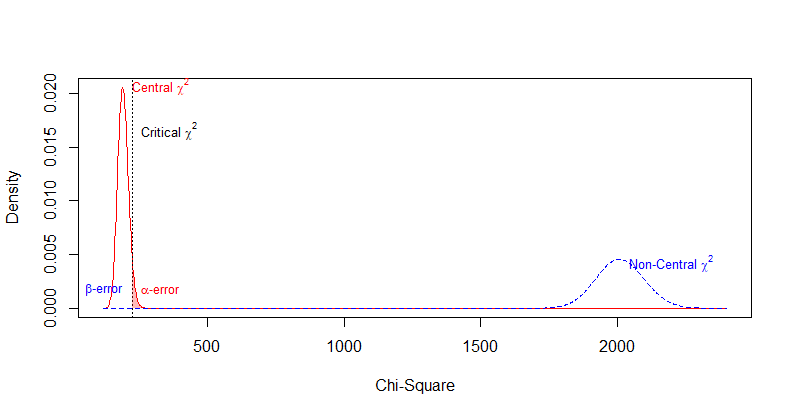 |
| --- |
| 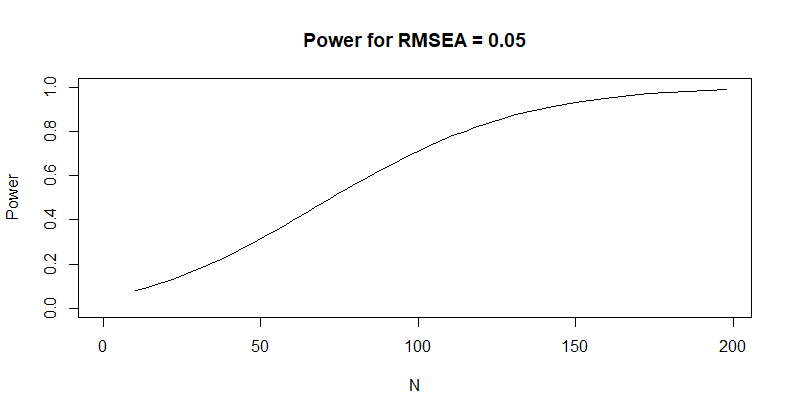 |
| **Supplementary Figure 1**. The top figure shows the chi-square expected distribution at different density values. The bottom figure shows the expected power as a function of degrees of freedom. As seen here, the expected power for a RMSEA=0.05 and df=190 is 0.99. |
